# Supplementary material for: Multiple risk factors are associated with an incremental increase in acute venous thromboembolism risk after total joint arthroplasty: A pearldiver cohort study
Source: PLoS One. 2024 Aug 9;19(8):e0308813. doi: 10.1371/journal.pone.0308813 (PMC11315297; doi:10.1371/journal.pone.0308813)
Supplement: S1 Table — (DOCX) [file pone.0308813.s001.docx]

**S1 table:** International Classification of Disease versions 9 and 10 and Current Procedural Terminology used to query risk factors.

| **Risk Factors** | **Code(s)** |
| --- | --- |
| Obesity | ICD-9-D-2780, ICD-9-D-27800, ICD-9-D-27801, ICD-9-D-27802, ICD-9-D-27803, ICD-10-D-E660:ICD-10-D-E669 |
| Cancer | ICD-9-D-140:ICD-9-D-17299, ICD-9-D-174:ICD-9-D-195899, ICD-9-D-200:ICD-9-D-20899, ICD-9-D-2386 ICD-10-D-C00:ICD-10-D-C2699, ICD-10-D-C30:ICD-10-D-C3499, ICD-10-D-C37:ICD-10-D-C4199, ICD-10-D-C43:ICD-10-D-C4399, ICD-10-D-C45:ICD-10-D-C5899, ICD-10-D-C60:ICD-10-D-C7699, ICD-10-D-C81:ICD-10-D-C8599, ICD-10-D-C88:ICD-10-D-C8899, ICD-10-D-CC90:ICD-10-D-C9799 |
| Congestive Heart Failure | ICD-9-D-39891, ICD-9-D-4280, ICD-9-D-4281, ICD-9-D-42820, ICD-9-D-42821, ICD-9-D-42822, ICD-9-D-42823, ICD-9-D-42830, ICD-9-D-42831, ICD-9-D-42832, ICD-9-D-42833, ICD-9-D-42840, ICD-9-D-42841, ICD-9-D-42842, ICD-9-D-42843, ICD-9-D-4289, ICD-10-D-I150:ICD-10-D-I159 |
| Varicose Veins | ICD-10-D-I8390, ICD-10-D-I8393 |
| Hormone Replacement Therapy | ICD-9-D-V074, ICD-10-D-Z79890 |
| Prior Arthroscopy Surgery | CPT-29861, CPT-29863, CPT-29870, CPT-29875, CPT-29876 |
| Tobacco Use | ICD-9-D-3051, ICD-9-D-98984, ICD-9-D-V1582, ICD-9-D-3051, ICD-10-D-F17220, ICD-10-D-F17221, ICD-10-D-F17223, ICD-10-D-F17228, ICD-10-D-F17229, ICD-10-D-F17290, ICD-10-D-F17291, ICD-10-D-F17293, ICD-10-D-F17298, ICD-10-D-F17299, ICD-10-D-Z720, ICD-10-D-F17200, ICD-10-D-F17201, ICD-10-D-F17203, ICD-10-D-F17208, ICD-10-D-F17209, ICD-10-D-F17210, ICD-10-D-F17211, ICD-10-D-F17213, ICD-10-D-F17218, ICD-10-D-F17219, ICD-10-D-F17220, ICD-10-D-F17221, ICD-10-D-F17223, ICD-10-D-F17228, ICD-10-D-F17229, ICD-10-D-F17290, ICD-10-D-F17291, ICD-10-D-F17293, ICD-10-D-F17298, ICD-10-D-F17299, ICD-10-D-Z716, ICD-10-D-Z720, ICD-10-D-Z87891 |
| Atrial fibrillation | ICD-9-D-42731, ICD-10-D-I481, ICD-10-D-I482, ICD-10-D-I4891 |
| Acute Myocardial Infarction | ICD-9-D-410:ICD-9-D-41099, ICD-9-D-412:ICD-9-D-41299, ICD-10-D-I21, ICD-10-D-I2199, ICD-10-D-I22:ICD-10-D-I2299, ICD-10-D-I252 |
| Hypertension | ICD-9-D-4010:ICD-9-D-4059 , ICD-10-D-I10:ICD-10-D-I159 |
| Type 2 Diabetes | ICD-9-D-25000, ICD-10-D-E119 |
