# Supplementary material for: Multiple risk factors are associated with an incremental increase in acute venous thromboembolism risk after total joint arthroplasty: A pearldiver cohort study
Source: PLoS One. 2024 Aug 9;19(8):e0308813. doi: 10.1371/journal.pone.0308813 (PMC11315297; doi:10.1371/journal.pone.0308813)
Supplement: Attachment — Submitted filename: Response to reviewers 5-29-2024.docx [file pone.0308813.s002.docx]

EDITOR’s COMMENTS AND REVIEW

Thank you so much for taking the time to review our manuscript and providing valuable feedback for improving the manuscript, the author’s replies are in blue font. Thanks

Journal Requirements:

1. When submitting your revision, we need you to address these additional requirements.

Please ensure that your manuscript meets PLOS ONE's style requirements, including those for file naming. The PLOS ONE style templates can be found at 
https://journals.plos.org/plosone/s/file?id=wjVg/PLOSOne_formatting_sample_main_body.pdf and 
https://journals.plos.org/plosone/s/file?id=ba62/PLOSOne_formatting_sample_title_authors_affiliations.pdf

Formatting and style requirements have been addressed throughout the manuscript.

2. We note that the grant information you provided in the ‘Funding Information’ and ‘Financial Disclosure’ sections do not match.

When you resubmit, please ensure that you provide the correct grant numbers for the awards you received for your study in the ‘Funding Information’ section.

Apologies for the mismatch. Grant number has been added. The study was funded by an internal institutional grant and hence does not create any conflicts of interest as all authors are employees of the institution. The grants are provided to encourage participation in research and the funder has no role in study design, data collection and analysis, decision to publish, or preparation of the manuscript.

3. Thank you for stating the following financial disclosure: 
Internal institutional funding from our hospital medical staff
 
Please state what role the funders took in the study.  If the funders had no role, please state: "The funders had no role in study design, data collection and analysis, decision to publish, or preparation of the manuscript." 
If this statement is not correct you must amend it as needed. 
Please include this amended Role of Funder statement in your cover letter; we will change the online submission form on your behalf.

Added to the manuscript as well as the cover letter

4. In the online submission form, you indicated that Aggregate data available with a subscription to PearlDiver
 
All PLOS journals now require all data underlying the findings described in their manuscript to be freely available to other researchers, either 1. In a public repository, 2. Within the manuscript itself, or 3. Uploaded as supplementary information.
This policy applies to all data except where public deposition would breach compliance with the protocol approved by your research ethics board. If your data cannot be made publicly available for ethical or legal reasons (e.g., public availability would compromise patient privacy), please explain your reasons on resubmission and your exemption request will be escalated for approval.

This study utilized PearlDiver, a proprietary database containing de-identified and anonymized medical insurance billing claims from 91 million orthopedic patients with Commercial, Medicare, and Medicaid insurance in the United States of America. Pearldiver does not provide patient level data publicly to protect patient privacy, all analysis are performed within the pearldiver platform and only aggregate results output is available which has been included in table format in the manuscript. If other researchers wish to replicate our results, they can do so by subscribing to pearldiver database and running the data query as we outline in methods.

5. Please include your full ethics statement in the ‘Methods’ section of your manuscript file. In your statement, please include the full name of the IRB or ethics committee who approved or waived your study, as well as whether or not you obtained informed written or verbal consent. If consent was waived for your study, please include this information in your statement as well.

 Included in paragraph 1 of methods

Additional Editor Comments:

I congratulate the esteemed authors for this important study. Venous thromboembolism (VT) remains a common and important complication after total joint arthroplasty surgeries. I think it was important that your study revealed a significantly increased incidence of VT, especially when there are three or more cumulative risk factors. Although the insurance billing database you mentioned in the limitation section has limitations, we kindly request you to expand the results section by providing more detailed information about risk factors in line with the suggestions of the reviewers and discuss them in the discussion section. Good luck.

5. Review Comments to the Author

Please use the space provided to explain your answers to the questions above. You may also include additional comments for the author, including concerns about dual publication, research ethics, or publication ethics. (Please upload your review as an attachment if it exceeds 20,000 characters)

**Reviewer #1**: Needs more elaboration on the risk factors , and almost the risk factors are not well categorized this format the paper would provides incomplete and even non specific information about DVT particularly and specifically about post operative DVT.

**Reviewer #2:** Acute venous thromboembolism(VTE) after total joint arthroplasty (TJA) was clinically very dangerous. Several risk factors are associated with it. t is very important to study the risk factors of the disease and carry out effective prevention, so this paper is of great significance and value to the clinic. The author's final conclusion was: persistent risk factors were associated with a higher incidence of postoperative VTE than transient risk factors. An incremental increase in risk was noted if three or more persistent risk factors were present. This is obvious and not particularly new. The article would be more meaningful if the various risk factors could be classified and the risk level or probability of each risk factor for VTE could be assessed.

**Reviewer #3:** This is an unprecedented and groundbreaking study that shows a risk score specific to total joint arthroplasty with respect to Acute VTE, which is good news for the field of total joint arthroplasty, where VTE is known to be common. The risk factors are expressed in terms of numbers, which is simplied and easy to understand. Independent factors have different risks, and I am not sure if this should be expressed in the same score. However, the simplified scores are extremely clear and easy to understand.

We greatly appreciate the esteemed editor and reviewers for your thoughtful review and suggestions for improvement.

As we noted in our manuscript, our study had several limitations secondary to the inherent limitations of utilizing a billing-based database. The Pearldiver database does not contain prescription data, there is a lack of clinical context and details on the status of persistent risks. For example, a diagnosis of diabetes is available, but appropriate laboratory values such as hemoglobin A1c are not available; hence, it is not feasible to determine whether diabetes is well-controlled or poorly controlled. Unfortunately, we are not able to elaborate on the risk factors beyond our current discussion in the manuscript.

**Despite these limitations, we believe that the robust sample size provides strength and validity to our findings regarding the cumulative impact of multiple concurrent risk factors.**

The clinical significance of our study is the addition of clarity to the current understanding of persistent and transient risk factors for patients undergoing lower extremity total joint arthroplasty in a large sample size (988,675 patients). Most individual, persistent risk factors increase patients' odds of acute VTE by 20-30%. Patients with multiple persistent risk factors incrementally increase their risk of acute VTE. Multiple persistent risk factors are associated with an incremental increase in VTE risk within the first three months after TJA. A strong association is noted when the number of persistent risk factors is three or more. To our knowledge, this is one of the first studies to report that the risk of VTE increases progressively with the presence of multiple risk factors and we hope that you will consider this suitable for publication
